# Deletion of UCP2 in iNOS Deficient Mice Reduces the Severity of the Disease during Experimental Autoimmune Encephalomyelitis

**DOI:** 10.1371/journal.pone.0022841

**Published:** 2011-08-08

**Authors:** Caroline Aheng, Nathalie Ly, Mairead Kelly, Saleh Ibrahim, Daniel Ricquier, Marie-Clotilde Alves-Guerra, Bruno Miroux

**Affiliations:** 1 Institut Cochin INSERM U1016, CNRS UMR8104, Faculté Paris Descartes, Paris, France; 2 Université Paris-Descartes, site Necker, INSERMU845, Paris, France; 3 Laboratoire de Biochimie A, Hôpital Necker-Enfants-Malades, Paris, France; 4 Genetics Group, Department of Dermatology, University of Luebeck, Luebeck, Germany; 5 Department of Immunology, University of Rostock, Rostock, Germany; 6 Laboratoire de Biologie Physico-Chimique des Protéines Membranaires, Institut de Biologie Physico-Chimique, CNRS UMR 7099, Université Paris-Diderot, Paris, France; University of Medicine and Dentistry of New Jersey, United States of America

## Abstract

Uncoupling protein 2 is a member of the mitochondrial anion carrier family that is widely expressed in neurons and the immune cells of humans. Deletion of *Ucp2* gene in mice pre-activates the immune system leading to higher resistance toward infection and to an increased susceptibility to develop chronic inflammatory diseases as previously exemplified with the Experimental Autoimmune Encephalomyelitis (EAE), a mouse model for multiple sclerosis. Given that oxidative stress is enhanced in *Ucp2−/−* mice and that nitric oxide (NO) also plays a critical function in redox balance and in chronic inflammation, we generated mice deficient for both *Ucp2* and *iNos* genes and submitted them to EAE. Mice lacking *iNos* gene exhibited the highest clinical score (3.4+/−0.5 p<0.05). Surprisingly, mice deficient for both genes developed milder disease with reduced immune cell infiltration, cytokines and ROS production as compared to *iNos*−/− mice.

## Introduction

Experimental Autoimmune Encephalomyelitis (EAE) is a demyelinating disease of the central nervous system (CNS) induced in susceptible mice strains by active priming with a specific myelin epitope (MOG_35–55_) combined with microbial adjuvant. Pathology of EAE is primarily characterized by a chronic peripheral immune response in secondary lymphoïd tissue. Consequent infiltration of lymphocytes and mononuclear cells in the CNS leads to the autoimmune destruction of the myelin sheath in brain and in the spinal cord and to neuronal cell death. The clinical expression of extensive demyelization can be observed as weakness of tail, moderate forelimb paraplegia and severe paraplegia. Because the pathological and the clinical aspects of EAE share similarities with human multiple sclerosis, a disease affecting more than 2.5 million people worldwide, EAE has been widely used as model to study genetic and immunological parameters favoring the development of the disease. Animal models have revealed different immunological mechanisms of action triggering axonal and neuronal lesions in spinal cord and in central nervous system. Depending of the model, auto-antibodies, macrophages or microglia cytotoxic products, class I MHC restricted cytotoxic cells or class II MHC restricted Th1 or Th17 cells are involved in the development of neuroinflammation. All these mechanism are, to some extent, relevant to human multiple sclerosis diseases showing the complexity of the disease (for review see [1]). In other hand, genes controlling energy metabolism and oxidative stress have been proposed to contribute to MS susceptibility by regulating the immune system activity and neuronal fitness. The roles of reactive oxygen species (ROS) in inflammation and in multiple sclerosis have been exemplified by several groups, suggesting that anti-oxidant therapies may re-enforce conventional immuno-therapies developed in the context of auto-immunity [2]. For instance, we have previously shown that mitochondrial Uncoupling protein 2 (UCP2) has a protective function during EAE [3]. Ucp2 mRNA is ubiquitous and the protein is readily detectable in tissues such as the spleen and lungs [4] and in immune cells like macrophages and T lymphocytes [5]. UCP2 has been shown to regulate ROS production in immune and in non-immune cells during pathological conditions such as atherosclerosis [6], type I diabetes [7], infections [5], cerebral ischemia [8] and EAE [3], thus protecting the organism from oxidative stress. Human genetic studies have confirmed these findings since the common G/A polymorphism at −866 upstream from *Ucp2* promoter is linked to atherosclerosis, diabetes [9] and multiple sclerosis [10]. More recently, we established a general link between chronic inflammation and *Ucp2* gene by showing that this common polymorphism contributes to eight other chronic inflammatory diseases, including rheumatoid arthritis, systemic lupus erythematosus, Wegener' granulomatosis, and psoriasis [11]. Several groups have suggested that UCP2 also plays a central role in the production of NO. Kizaki and colleagues showed that UCP2 expression in the macrophage cell line Raw264 induces a marked reduction of ROS and *iNos* gene expression upon lipopolysaccharides (LPS) treatment [12]. Conversely, immune challenge with LPS led to increased expression of iNOS protein and enhanced NO production in *Ucp2* deficient mice [13]. Emre et al. have proposed that transient decreases of UCP2 expression at an early stage of inflammation increases mitochondrial ROS production, which in turn, activates the mitogen-activated protein kinase pathway thus leading to iNOS protein expression and enhanced inflammation [14]. Production of NO is significantly increased in CNS lesions, MS patients' blood and urine but also in animals with EAE as revealed by paramagnetic resonance spectroscopy analysis [15]. NO has long been considered as a potential contributor to EAE pathogenesis because highly reactive derivatives of NO such as peroxynitrites induce tissue damage and compromise the blood brain barrier permeability [16]. However, genetic deletion of *iNos* gene was associated with an increased susceptibility of mice to EAE suggesting an important down-regulatory role of NO on the immune system both in the periphery and target tissue [17], [18]. Indeed, NO can inhibit T lymphocyte proliferation and modulate the Th1/Th2 cytokines balance by favoring apoptosis or necrosis of autoreactive Th1 cells [19], [20]. Considering the dual effect of NO in EAE i.e., inhibition of autoreactive T cells and tissue damaging ability and the protective effect of UCP2 in EAE, we generated mice deficient for both genes and submitted them to EAE. Clinical scores and immune parameters demonstrated that (*Ucp2-iNos*) deficient mice developed a milder disease associated with decreased ROS production by macrophages.

## Results

### Clinical parameters after MOG immunization

**Figure 1:**
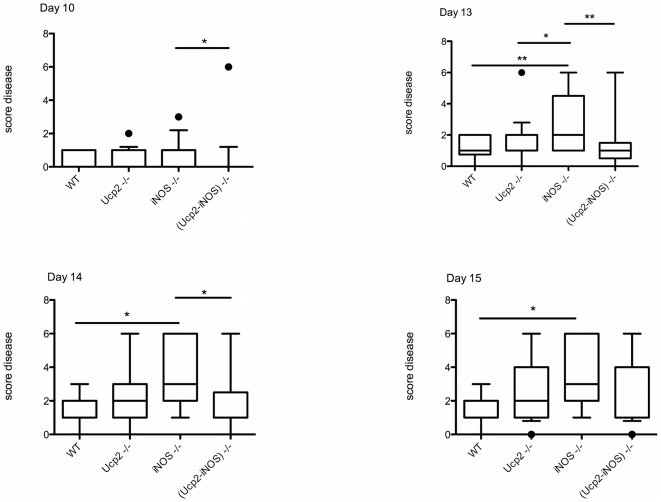
Score disease of mice upon EAE induction. EAE was induced in 7–10 weeks of age mice by subcutaneous injection with 150 µg MOG_35–55_ peptide in presence of Freund adjuvant and Pertussis toxin (see Material and Methods). Clinical symptoms were scored as follows: 0, normal; 1, weak/flaccid tail; 2, waddle; 3, moderate paraparesis; 4, severe paraparesis; 5, tetraparesis; 6, moribund. The mean clinical score was determined as the average score of all animals for a given genotype at days 10, 13, 14 and 15. Statistical significance was determined by one way ANOVA and free parametric Kruskal-Wallis test. *, *P*<0.05; **, *P*<0.01; ***, *P*<0.001.

As previously reported [3], *Ucp2* deficient mice exhibited an increased score diseases during EAE. However, in this study, score diseases between *Ucp2* deficient mice and control mice were not significantly different (Fig. 1), while in our previous study a significant difference was observed all over the induction phase. This is probably due to the reduced number of mice (18 instead of 40) used in this study (Table 1). Significant differences in disease severity between *iNos* deficient mice and the other genotypes were observed as from day 10. At days 13, 14 and 15, mean clinical scores were significantly increased in *iNos* deficient mice as compared to wild type mice (Fig. 1). Interestingly deletion of *Ucp2* gene in *iNos* deficient mice significantly delayed the onset of the disease (p = 0.0003) and decreased the severity of the diseases observed in the *iNos* deficient mice at day 10, 13 and 14 (Fig. 1, Table 1). Other clinical parameters, i.e incidence of the disease, mortality, were not significantly affected (Table 1).

**Table 1:** Clinical parameters of the mice during Experimental Autoimmune Encephalomyelitis.

	N^1^	Mean clinical score			Mean day of onset	Incidence	Mortality
						(%)	(%)
Vogler et al.		Day 13	Day 14	Day 15			
Ucp2+/+	25			1.7±0.2	11.5±0.8	75	
Ucp2−/−	40			2.9±0.2	13.0±0.6	75	
This study		Day 13	Day 14	Day 15			
*Ucp2+/+*	18	1.1±0.2	1.4±0.2	1.4±0.2	11.9±0.7	90	16.6
*Ucp2*−/−	17	1.4±0.3	2.3±0.4	2.5±0.4	11.8±0.6	89.4	17.6
*iNos*−/−	17	2.9±0.5	3.4±0.5	3.5±0.5	10.5±0.6	94.4	29.4
(*Ucp2-iNos*)−/−	17	1.4±0.4	1.7±0.4	2.4±0.5	13.5±0.3	89.5	17.6

1 N = number of mice; Data represent means ± SEM.

### Immune cell infiltration and cytokine production in the central nervous system upon EAE induction

**Figure 2:**
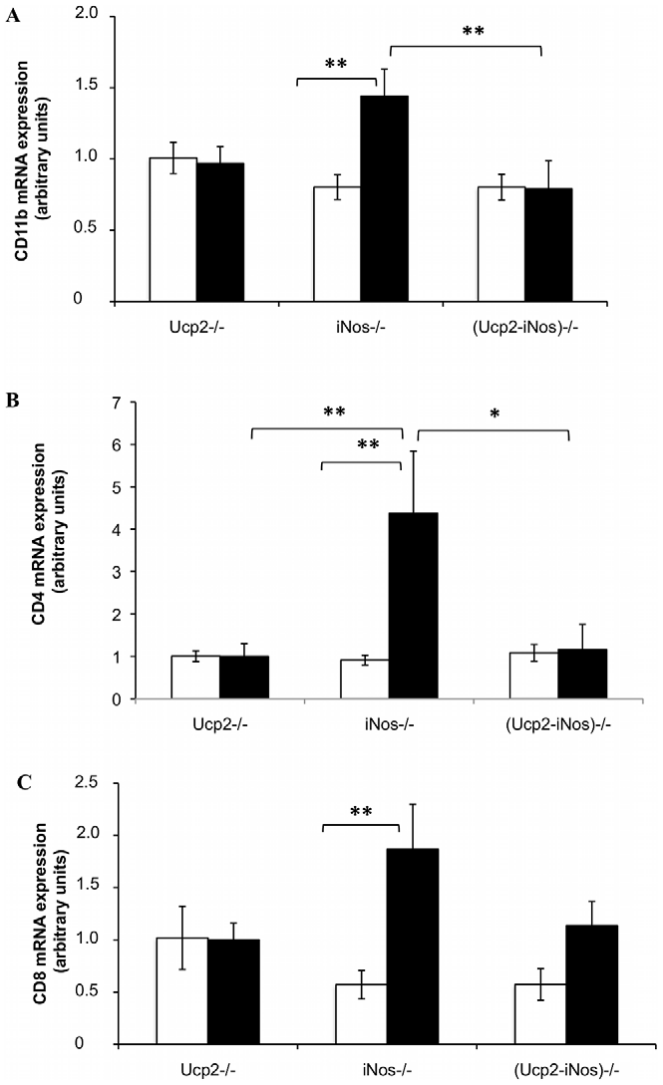
Infiltration of immune cells in the central nervous system. Both brain and spinal cord from MOG immunized mice of all genotypes were collected at days 10 (white) and day 14 (grey). Levels of mRNAs specific for the monocyte cell marker CD11b (A), and for the T cell markers CD8 (B) and CD4 (C) were assessed by real time quantitative RT-PCR. The data are expressed as the relative mean ± SEM of 9–12 mice per genotype using *GADPH* as the housekeeping gene. Statistical significance was determined by ANOVA. *, *P*<0.05; **, *P*<0.01; ***, *P*<0.001.

**Figure 3:**
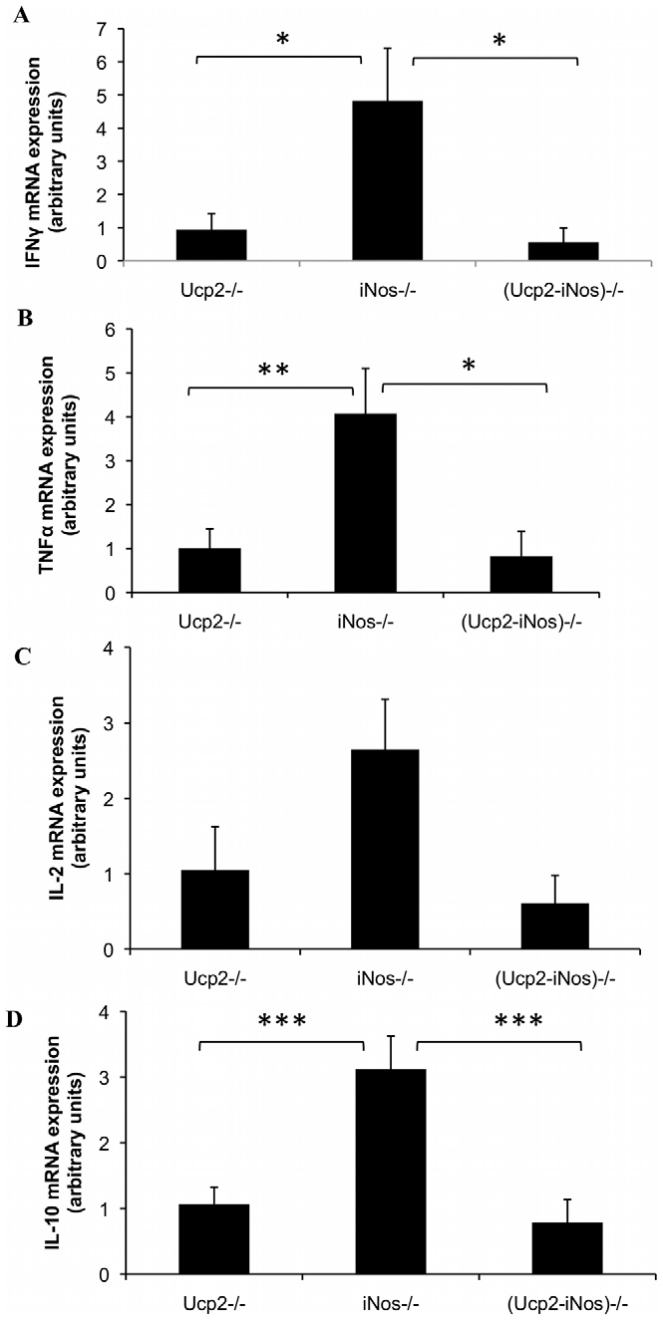
Relative expression of cytokines mRNA in CNS homogenates. Fourteen days after EAE induction, brains and spinal cords were collected and levels of mRNAs were assessed by real time quantitative PCR as described in *Materials and Methods*. Data represent mean ± SEM of 7–12 mice per strain. *, *P*<0.05; **, *P*<0.01; ***, *P*<0.001.

To evaluate the loss of the BBB integrity and the consequent infiltration of activated immune cells, mRNA expression levels of monocytes/macrophages cell marker CD11b (Fig. 2A), and the T cell markers CD4 (Fig. 2B) and CD8 (Fig. 2C) were assayed by real-time quantitative RT-PCR. At day 14 after immunization with the MOG peptide, the *iNos* deficient mice showed increased accumulation of CD11b mRNA (p<0.01 versus (*Ucp2-iNos*) deficient mice) and of CD4 mRNA (p< = 0.05 versus (*Ucp2-iNos*) deficient mice). No differences were observed in the accumulation of CD8 mRNA in the CNS of the different strains. Cytokines mRNA levels were measured in the inflamed CNS at day 14. The *iNos* deficient mice exhibited increased expression of the Th1 cytokines IFNγ (Fig. 3A), TNFα (Fig. 3B) and the Th2 cytokine IL-10 (Fig. 3D) when compared to *Ucp2* deficient mice, and (*Ucp2-iNos*) deficient mice. Changes in Il-2 levels (Fig. 3D) were not statistically significant.

### Oxidative stress analysis

**Figure 4:**
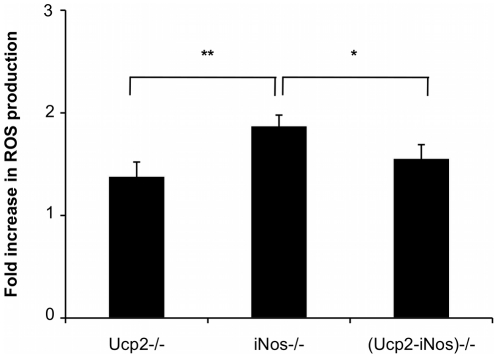
ROS production in macrophages from *Ucp2−/−*, *iNos−/−*, and *(Ucp2-iNos)−/−* deficient mice. Peritoneal macrophages were obtained at day 10 after MOG immunization and stimulated *in vitro* with 500 ng/ml phorbol merystil acetate during 1 hour as described in *Materials and Methods*. Data represent mean ± SEM from 5–13 mice per strain. Student's *t* test was performed to determine the statistical significance of the differences between *Ucp2−/−*, *iNos−/−* and *(Ucp2-iNos)−/−* mice. *, *P*<0.05; **, *P*<0.01.

**Figure 5:**
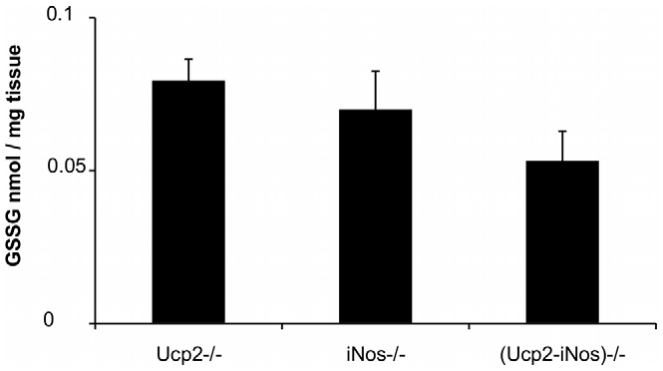
Oxidized glutathione in the CNS. Oxidized glutathione was assayed 14 days after EAE induction, as described in experimental procedures, in the CNS from *Ucp2−/−*, *iNos−/−* and *(Ucp2-iNos)−/−* deficient mice. Data represents means ± SEM of 4–5 mice per genotype.

Reactive Oxygen Species (ROS) are key mediators that can regulate the intensity of an immune response. In order to assess the oxidative status of the immune system during EAE, we measured the ability of peritoneal exudates from MOG immunized *Ucp2*−/−, *iNos*−/− and (*Ucp2-iNos*) deficient mice to produce ROS *in vitro* 10 days after EAE induction (Fig. 4). *In vitro* stimulation with PMA generated increased production of ROS by *iNos* deficient macrophages compared to *Ucp2* and (*Ucp2-iNos*) deficient macrophages. Oxidative stress in the brain and in the spinal cord of mice was also estimated by measuring the levels of GSH and GSSG. Levels of GSH and of GSSG were not significantly different between strains (data not shown) but GSSG content had a tendency to decrease in the CNS of *(Ucp2- iNos)* deficient mice (Fig. 5).

## Discussion

We have shown that deletion of *iNos* gene induces a more severe disease than the deletion of *Ucp2* gene. In addition, the inactivation of both *Ucp2* and *iNos* genes is protective in EAE progression as demonstrated by delayed onset of the disease, reduced disease severity, decreased immune cell infiltration and cytokine production observed in (*Ucp2-iNos*) deficient mice.

**Figure 6:**
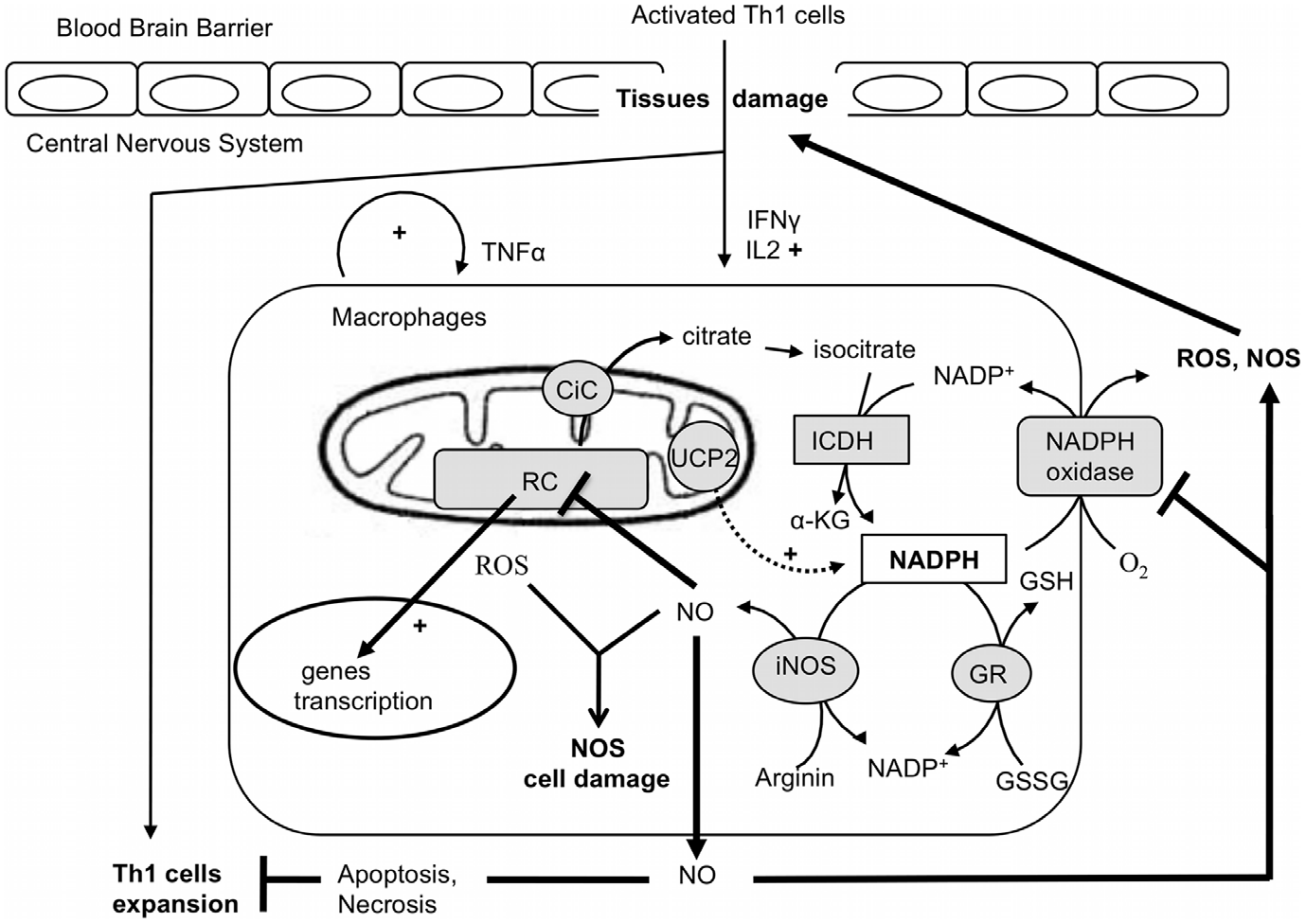
The redox balance in macrophages during inflammation induced by EAE. Once activated, Th1 cells enter the CNS and produce proinflamatory cytokines (IFNγ, Il2) that stimulate resident macrophages. Inflammation is further increased with the autocrine production of TNFα by macrophages. At the cellular level, ROS production stimulates the expression of proinflammatory genes such as the NO synthase gene. In one hand, nitric oxide inhibits the respiratory chain (RC), which, in turn, increases the mitochondrial ROS production and consequently oxidative cell damage. In the other hand, NO prevent Th1 cell expansion and inhibits the NAPH oxidase enzyme, an essential ROS producer that trigger tissue damage and brain blood barrier destruction. At the molecular level, NADPH plays a pivotal function in the redox balance as substrate of the NADPH oxidase, the NO sytnhase (iNOS) and the gluthation reductase (GR). In this context, mitochondrial carriers, including UCP2, could modulate the availability of this substrate. For instance the citrate carrier supplies an additional source of cytosolic NADPH by exporting citrate that is further oxidized into alpha keto glutarat (α-KG) by the cytosolic isocitrate dehydrogenase (ICDH).

Considering the above results and data available in literature, we propose the following scenario (Fig. 6). Activated T cells enter the CNS and they are reactivated by microglia to produce pro-inflammatory cytokines, IFNγ and IL-2. Production of IFNγ by T cells within the CNS activates microglia and resident macrophages to produce TNFα, NO and ROS. Whereas TNFα allows recruitment of macrophages in the inflamed CNS, NO readily diffuses through the mitochondrial outer and inner membranes where it reversibly inhibits cellular respiration by competing with oxygen at the level of the cytochrome oxidase [23], thus triggering a reduced state of the respiratory chain that favors O_2_.- formation (Fig. 6). When NO is extensively produced, as is the case in *Ucp2*−/− macrophages [13], it may exert cytotoxic effects on complex I and II of the respiratory chain [24] and thus triggering ROS production by mitochondria and peroxynitrite formation as previously observed in atherosclerotic plaque of *Ucp2*−/− mice [6]. On the other hand, NO is also known to inhibit the NADPH oxidase [25], a major player in macrophage ROS production and an essential gene for the development of EAE since NADPH oxidase deficient mice are resistant to disease development [26]. NO also acts as a feedback loop to inhibit expansion of autoreactive T cells and therefore participate to the resolution of the inflammation (Fig. 6). In *iNos* deficient mice NO is no longer produced and this may therefore increase the infiltration and expansion of autoreactive CD4 cells. Interestingly, our study shows that inactivation of both *Ucp2* and *iNos* genes leads to a less severe disease. The decreased ability of (*Ucp2-iNos*) deficient macrophages to produce ROS at an early stage of the disease (day 10) may represent the primary mechanism that confers protection to double knockout mice.

Despite the fact that UCP2 shares 60% homology with Uncoupling protein 1, a proton transporter that uncouples ATP synthesis from mitochondrial respiration in brown adipose tissue, UCP2 does not seem to ensure such an uncoupling activity *in vivo* [27]. Several studies [28]–[30] have shown that UCP2 is not a relevant proton transporter as UCP1 is, but rather an anion transporter. UCP2 protein has recently been proposed as pyruvate exporter that would transiently change the balance between glucose and free fatty acid oxidation, thus impacting ROS production and glucose sensing [31]. This “pyruvate hypothesis” can also impact the redox cytosolic state of the macrophage [32]. For instance, Nuebel et al. reported a decrease of the NADH/NAD^+^ ratio in bone marrow derived macrophages isolated from *Ucp2−/−* mice [28]. Moreover the citrate carrier, another member of the mitochondrial carrier family, has been proposed to play an anaplerotic function by regulating the cytosolic pool of NADPH. Indeed, by exporting citrate produced in the mitochondrial matrix to the cytosol, the citrate carrier provides substrate for the cytosolic NADP^+^ isocitrate dehydrogenase enzyme, which generates NADPH [33] that is used by the redox machinery including the NADPH oxidase, the iNOS synthase and the glutathione reductase enzymes. Therefore, the cell redox status results from the level of expression and activity of those enzymes and from the availability of their substrates (Fig. 6). This “NADPH hypothesis” need to be further explored in (*Ucp2*) deficient mice, because it could explain the decreases in ROS production by macrophages in *(Ucp2-iNOS)* deficient mice. Finally, our study illustrates the interplay between mitochondrial ROS and cytosolic NO productions in inflammation and highlights the importance of the mitochondrial metabolism in the redox state of immune cells.

## Methods

### Animals and induction of EAE

Studies on mice were performed in agreement with the institutional CNRS guidelines defined by the European Community guiding principles and by the French decree N°87/848 of October 19, 1987. Authorization to perform animal experiments was given by the French ministry of Agriculture, fisheries and food (A92580 issued February 2 1994 and 92–148 issued May 14, 2002). All protocols on mice were declared to the “Landesveterinär- und Lebensmitteluntersuchungsamt Mecklenburg

Vorpommern » gouvernement office and approved under the permit number No: LVL-MV 310-4/7221.3-1.1-024/2. All chemicals were obtained from Sigma-Aldrich (Saint Louis, MO). Double knock out animals were obtained by crossing *Ucp2*−/− and *iNos*−/− deficient mice on the EAE- susceptible C57BL/6 background. The (*Ucp2−/−*) mice have been previously described [21], [22]. The (*iNos−/−*) mice were obtained from the Jackson laboratory (Bar Harbor, ME). The F1 generation produced *(Ucp2+/− iNos+/−)* heterozygote mice. The F2 generation produced *(Ucp2−/− iNos+/+) mice*, *(Ucp2+/+ iNos−/−)* and (*Ucp2−/− iNos*−/−) mice. All mice were fed with sterile food and water ad libitum and kept in our animal facility at the Faculté de Médecine Necker in Paris. Inactivation of both genes was controlled by PCR analysis as previously described [17], [21]. EAE was induced in 7–10 weeks of age mice by subcutaneous injection with 150 µg of myelin oligodendrocyte glycoprotein (MOG _35–55_) peptide (Genosphere Biotechnologies, Paris, France) dissolved in 50 µl of distilled water and emulsified with an equal volume of incomplete Freund adjuvant complemented with 4 mg/ml *Mycobacterium tuberculosis* (Difco Laboratories, Detroit, MI). Mice also received an additional peritoneal injection of 400 ng of Pertussis toxin on day 0 and day 2. Clinical symptoms were scored as follows: 0, normal; 1, weak/flaccid tail; 2, waddle; 3, moderate paraparesis; 4, severe paraparesis; 5, tetraparesis; 6, moribund. The mean clinical score was determined as the average score of all animals for a given genotype at a given day. Mice showing no clinical symptoms at all were excluded from the analysis.

### ROS production by peritoneal macrophages

Peritoneal macrophages were obtained from diseased animals at days 10, 14 and 30 after EAE induction. They were subsequently washed twice in 10 ml ice-cold PBS. Red blood cells were lysed with a 0.83% NH_4_CI Tris-Buffered solution. Remaining cells were then counted and cultured in 96 well plates at a density of 10^5^ cells per well and allowed to adhere for 2 hours at 37°C. Non adherent cells were eliminated by washing the plates twice with PBS. Adherent macrophages were stimulated with 500 ng/ml PMA in 100 µL of 1 mg/ml NBT dissolved in RPMI 1640. After one-hour stimulation, the plates were centrifuged and supernatant was discarded. The formazan precipitate was washed and dissolved with an equal volume of DMSO and 2 M KOH solution as previously described [21]. Absorbance was read at 630 nm.

### Real-Time Quantitative RT-PCR

To evaluate cytokine expression and immune cells infiltration, both spinal cord and brain were collected from diseased mice at days 10 and 14 after EAE induction. The tissues were individually grinded in phosphate buffer using a nylon mesh to separate cells. The suspension was centrifuged at 3000 g for 10 minutes and the resulting cell pellet was resuspended in 1 ml TRIzol (Invitrogene, Carlsbad, CA) for RNA isolation according to manufacturer's instructions. First strands of cDNA were synthesized from 1 µg total RNA by using iScript cDNA synthesis kit (Bio-Rad, Hercules, CA) and then subjected to PCR using specific primers in a Bio-Rad iCycler iQ real-time detection system. Data were calculated on the basis of threshold cycle (C_T_) for the target gene and the reference gene GADPH according to the following formula: 2 Δ(C_T(GADPH)_ - C_T(Target)_). Oligonucleotide sequences used were IL-4 forward 5′-GGT CTC AAC CCC CAG CTA GT-3′; IL-4 reverse 5′-GCC GAT GAT CTC TCT CAA GTG AT-3′; IL10 forward 5′-CTG GAC AAC ATA CTG CTA ACC G-3′; IL-10 reverse 5′-GGG CATC ACT TCT ACC AGG TAA-3′; TNFα forward 5′-CCC TCA CAC TCA GAT CAT CTT CT-3′; TNFα reverse 5′-GCT ACG ACG TGG GCT ACA G-3′; IFN γ forward 5′-GAA CTG GCA AAA GGA TGG TGA-3′; IFNγ reverse 5′-TGT GGG TTG TTG ACC TCA AAC-3′; CD4 forward 5′-TCA CCT GGA AGT TCT CTG ACC-3′; CD4 reverse 5′-GGA ATC AAA ACG ATC AAA CTG CG-3′; CD8 forward 5′-CAT CCT GCT TCT GCT GGC ATT-3′; CD8 reverse 5′-TGG GCG CTG ATC ATT TGT GAA A-3′; CD11b forward 5′- ATG GAC GCT GAT GGC AAT ACC-3′; CD11b reverse 5′-TCC CCA TTC ACG TCT CCC A-3′; GAPDH forward 5′-TGA CCA CAG TCC ATG CCA TC-3′; GADPH reverse 5′- GAC GGA CAC ATT GGG GGT AG-3′.

### Determination of oxidative stress in CNS

Fresh brain and spinal cord tissues were collected from diseased animals (day 14) and minced in TES buffer (10 mM Tris, pH 7.5, 1 mM EDTA, 250 mM sucrose). Thirty microliters of the resulting homogenate was sonified for 30 seconds. Ten microliters of 10% metaphosphoric acid and 170 µl of 10 mM NaH_2_PO_4_, pH 2.7 were subsequently added to each sample, thoroughly mixed and allowed to incubate on ice for 10 minutes for deproteinisation. After incubation, samples were centrifuged for 5 minutes at 15 000 g and supernatant was kept on ice until analysis. Reduced (GSH) and oxidized glutathione (GSSG) were simultaneously determined by high-performance liquid chromatography with electrochemical detection. Separation was performed on an Uptisphere column (ODB 250×4.6 mm, 5 µm; Interchim, Montluçon, France) using a mobile phase containing 10 mM NaH_2_PO_4_, 1.25% methanol, pH 2.7. Analytical cell potentials were set at 400 mV for E1 and 900 mV for E2. The retention times of GSH and GSSG were 5.4 and 12.6 min, respectively.

### Statistical analysis

Evaluation of statistical differences between genotypes was determined by Anova and Bonferroni's multiple comparaison test. In all cases, *P*<0.05 were considered significant.
